# Effect of Annealing Temperature and Oxygen Flow in the Properties of Ion Beam Sputtered SnO_2−x_ Thin Films

**DOI:** 10.3390/ma8085243

**Published:** 2015-08-14

**Authors:** Chun-Min Wang, Chun-Chieh Huang, Jui-Chao Kuo, Dipti Ranjan Sahu, Jow-Lay Huang

**Affiliations:** 1 Department of Materials Science and Engineering, National Cheng Kung University, No. 1, University Road, Tainan 701, Taiwan; E-Mails: n5896114@mail.ncku.edu.tw (C.-M.W.); jckuo@mail.ncku.edu.tw (J.-C.K.); jlh888@mail.ncku.edu.tw (J.-L.H.); 2 Department of Electrical Engineering, Cheng Shiu University, No. 840, Chengcing Road, Niaosong Township, Kaohsiung 833, Taiwan; 3 Amity Institute of Nanotechnology, Amity University, Sector 125, Noida, India; E-Mail: dsahu@amity.edu; 4 Department of Chemical and Materials Engineering, National University of Kaohsiung, No. 700, Kaohsiung University Road, Nan-Tzu District, Kaohsiung 811, Taiwan; 5 Center for Micro/Nano Science and Technology, National Cheng Kung University, Tainan 701, Taiwan; 6 Research Center for Energy Technology and Strategy, National Cheng Kung University, Tainan 701, Taiwan

**Keywords:** SnO_2_, transparent conductive oxide (TCO), oxygen flow ratio, annealing

## Abstract

Tin oxide (SnO_2−x_) thin films were prepared under various flow ratios of O_2_/(O_2_ + Ar) on unheated glass substrate using the ion beam sputtering (IBS) deposition technique. This work studied the effects of the flow ratio of O_2_/(O_2_ + Ar), chamber pressures and post-annealing treatment on the physical properties of SnO_2_ thin films. It was found that annealing affects the crystal quality of the films as seen from both X-ray diffraction (XRD) and transmission electron microscopy (TEM) analysis. In addition, the surface RMS roughness was measured with atomic force microscopy (AFM). Auger electron spectroscopy (AES) analysis was used to obtain the changes of elemental distribution between tin and oxygen atomic concentration. The electrical property is discussed with attention to the structure factor.

## 1. Introduction

The most widely-used materials for transparent conductive oxide (TCO) thin films are zinc oxide (ZnO), tin oxide (SnO_2_) and indium oxide (In_2_O_3_). SnO_2_ thin films have been developed and applied in different fields, such as solar cells, gas sensors and light-emitting diodes (LEDs) [1,2,3,4,5] because they have the advantages of their low electrical resistance and high optical transparency in the visible range of the electromagnetic spectrum. Furthermore, they are inexpensive and also stable against thermal and chemical attacks at high temperature.

A number of studies have been reported on the effect of the oxygen gas ratio [6], substrate temperature and type, annealing temperature [7,8,9] and doping elements [10,11,12] on improving the structural, electrical and optical properties of the SnO_2_ thin film in the past few decades. The thin films of pure or doped SnO_2_ can be prepared by different deposition techniques, including sol-gel-dip coatings [13], metal-organic chemical vapor deposition (MOCVD) [14,15], chemical bath deposition (CBD) [16], spray pyrolysis [17], electron beam evaporation [18] and sputtering [19,20,21,22]. However, it is necessary to carry out either a heat treatment of substrates during the deposition process or the post-annealing procedure to obtain low resistivity and to retain high transmittance. Here, the ion beam sputtering (IBS) technique was applied to achieve high quality films, because of its high energy of incoming ions and the low deposition rate.

In general, SnO_2_ thin films have defects, such as oxygen vacancies [23], which act as donors in the SnO_2_ matrix and increase the electron density in the conduction band. This is called the n-type conduction. The formation of excess oxygen vacancies results in decreasing film quality. Thus, increasing the conductivity of SnO_2−x_ lies in a narrow range of oxygen pressure [24]. The post-heat treatment reduces residual stress and the lattice mismatch to obtain good electrical conductivity [25].

In the present work, we investigated the effects of the flow ratio of O_2_/(O_2_ + Ar), chamber pressures and post-annealing treatment on the physical properties of SnO_2_ thin films.

## 2. Results and Discussion

### 2.1. Structural and Morphological Properties

**Figure 1:**
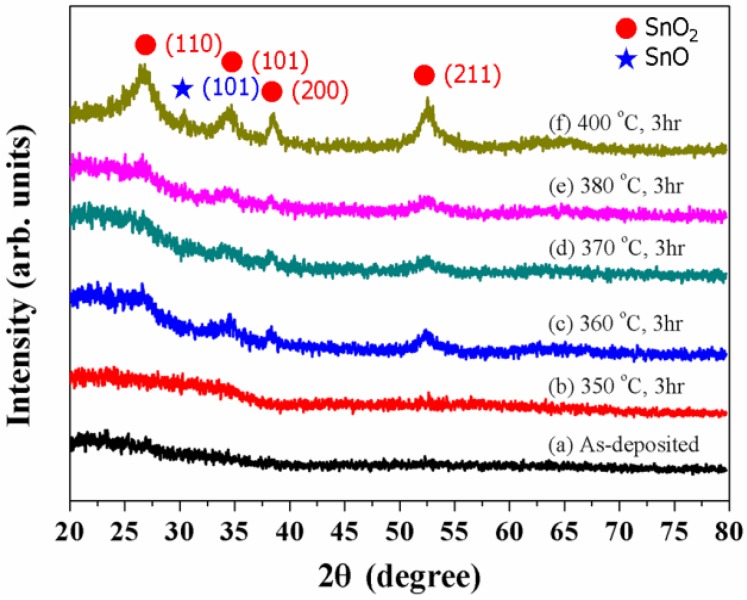
XRD patterns of SnO_2−x_ thin films (**a**) as-deposited and annealed for 3 h at (**b**) 350 °C; (**c**) 360 °C; (**d**) 370 °C; (**e**) 380 °C and (**f**) 400 °C at 4.7 × 10^−2^ Pa.

Figure 1 shows typical XRD patterns of the as-deposited and annealed SnO_2−x_ films where they were prepared at a flow ratio of 0.5 of O_2_/(O_2_ + Ar) at 4.7 × 10^−2^ Pa. The structures of the as-deposited and the annealed SnO_2−x_ films deposited at 4 × 10^−2^ Pa were almost the same as that prepared. It is observed that the films as-deposited and annealed at 350 °C were the typical amorphous structure, as indicated in Figure 1a,b. However, SnO_2−x_ films annealed at 360 °C have crystallization without preferred orientations, as shown in Figure 1c. A similar result was reported by Wulff *et al.* [26] for ITO thin films. Further, the {101} peak of SnO phase appeared at 400 °C, as shown in Figure 1f, which was also reported by Choe *et al.* [9].

**Figure 2:**
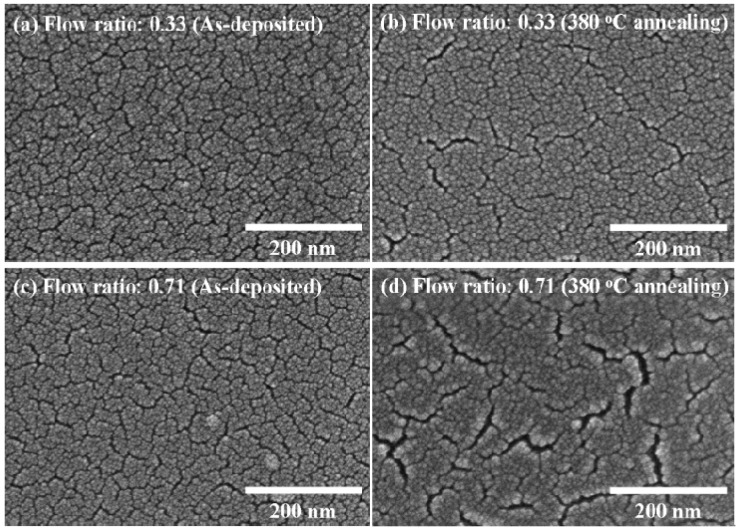
FE-SEM micrographs of the SnO_2−x_ thin films prepared at various flow ratios and a working pressure of 4 × 10^−2^ Pa. The flow ratio of 0.33 of O_2_/(O_2_ + Ar) (**a**) for as-deposited films; (**b**) 380 °C annealing and the flow ratio of 0.71 of O_2_/(O_2_ + Ar) (**c**) for as-deposited films; (**d**) 380 °C annealing.

Figure 2 shows the surface morphology of SnO_2−x_ films prepared with low and high flow ratios of 0.33 and 0.71 at 4 × 10^−2^ Pa. It is observed that the as-deposited films have similar and uniform surface morphologies. However, cracks are found at a high O_2_/(O_2_ + Ar) flow ratio of 0.71 after 380 °C annealing.

**Figure 3:**
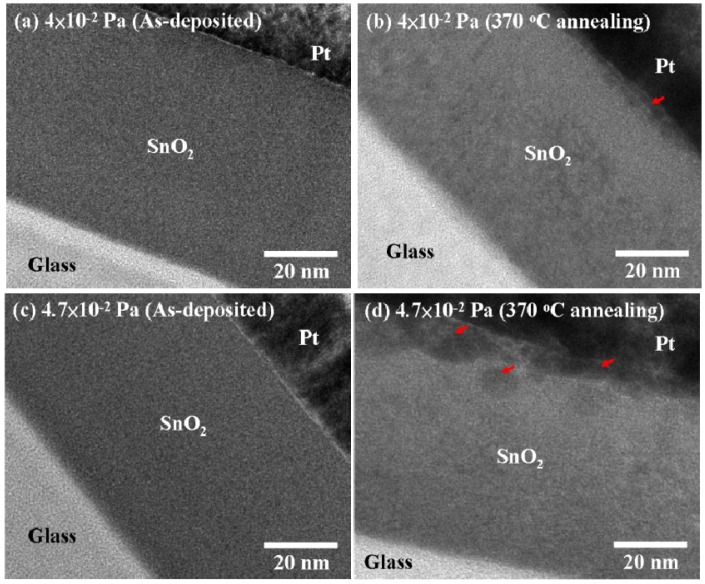
The cross-section images of bright field high-resolution TEM analysis at the same flow ratio of 0.71 of O_2_/(O_2_ + Ar) and a working pressure of 4 × 10^−2^ Pa (**a**) for as-deposited films; (**b**) 370 °C annealing and a working pressure of 4.7 × 10^−2^ Pa (**c**) for as-deposited films; (**d**) 370 °C annealing.

In addition to SEM analysis, TEM was employed to characterize the cross-section of SnO_2−x_ films. The as-deposited films with a high flow ratio of 0.71 of O_2_/(O_2_ + Ar) at 4 × 10^−2^ and 4.7 × 10^−2^ Pa reveal very high adherence and a smooth and uniform surface, as shown in Figure 3a,c. After annealing at 370 °C for 3 h, the films show looser, thinner interfaces than as-deposited films, because they were cracked and have enhanced large spaces between grains after high temperature Ar annealing, as shown in Figure 3b,d.

**Figure 4:**
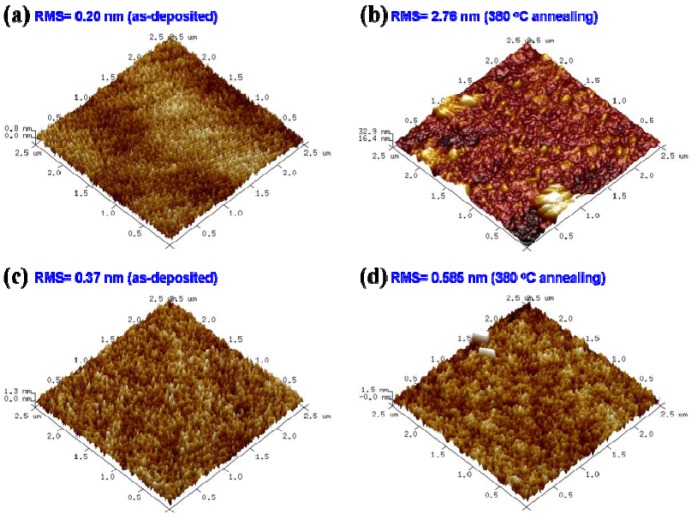
3D AFM images with a scan area of 2.5 μm × 2.5 μm: (**a**) as-deposited; (**b**) 380 °C annealing SnO_2−x_ thin films at the flow ratio of 0.5 of O_2_/(O_2_ + Ar) and a working pressure of 4 × 10^−2^ Pa. (**c**) As-deposited; (**d**) 380 °C annealing SnO_2−x_ thin films at the flow ratio of 0.5 of O_2_/(O_2_ + Ar) and a working pressure of 4.7 × 10^−2^ Pa.

The AFM images that illustrate the surface topology and the root mean square (RMS) surface roughness of SnO_2−x_ films before and after 380 °C annealing are shown in Figure 4. It is clear that the surface morphology of the as-deposited film and 380 °C annealing at the flow ratio of 0.5 of O_2_/(O_2_ + Ar) do not significantly change at a working pressure of 4.7 × 10^−2^ Pa, as shown in Figure 4c,d. However, the surface morphology of the film changes dramatically from Figure 4a to Figure 4b. The surface roughness of the as-deposited film at 4 × 10^−^^2^ Pa is 0.20 nm. The film annealed at 380 °C shows an increase of surface roughness from 0.20 nm to 2.76 nm. The increase in film roughness at a working pressure of 4 × 10^−2^ Pa can be explained with surface melt after 380 °C annealing. However, it does not have an obvious structure transformation at a working pressure of 4.7 × 10^−2^ Pa.

### 2.2. Electrical Properties

**Figure 5:**
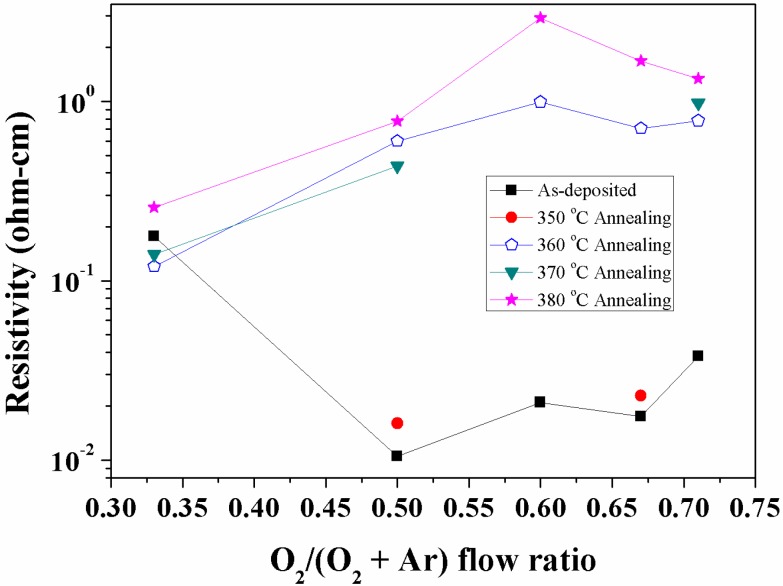
The variation of the electrical resistivity of SnO_2−x_ thin films with various flow ratios of O_2_/(O_2_ + Ar) at the annealing temperature from 350 °C to 380 °C and a working pressure of 4.7 × 10^−2^ Pa.

Figure 5 shows the variation of electrical resistivity of SnO_2_ thin films with various flow ratios of O_2_/(O_2_ + Ar) at the annealing temperature from 350 °C to 380 °C and a working pressure of 4.7 × 10^−2^ Pa.

The as-deposited films show the lowest resistivity of 1.05 × 10^−2^ Ω cm at the flow ratio of 0.5. A strong dependence of resistivity on the oxygen flow ratio was observed. It is observed that the resistivity increased about two orders of higher magnitude at 360 °C than that of the as-deposited films, except at the flow ratio of 0.33. This is due to the change in the structure from amorphous to crystallization and mainly the decrease of carrier concentration with a serious crack formation at a higher flow ratio, as depicted by SEM and TEM analyses. Shanthi *et al.* [27] reported that the chemisorbed oxygen removes oxygen vacancies at high temperature annealing and increases chemisorbed oxygen at the surfaces and grain boundaries, which results from further lowering of free electrons. The degree of reduction in carrier concentration was high for SnO_2_ deposited at a high flow ratio. At the oxygen flow ratio of 0.3, the initial decrease in resistivity at the annealing temperature from 360 °C to 370 °C is due to the number of oxygen vacancies and excess metal ions arising from the non-stoichiometry, which was also reported by Ku *et al.* [28]. The flow ratio of 0.6 has the highest resistivity due to its lowest mobility or carrier concentration at high and low working pressure after annealing. Williams and Ho [29,30] reported the conductivity performance of SnO_2_ sensors, which were very a sensitive probe of changes in surface chemistry due to electrons transfer, such as the parameters of oxygen partial pressure and crystallite size, *etc.*

**Figure 6:**
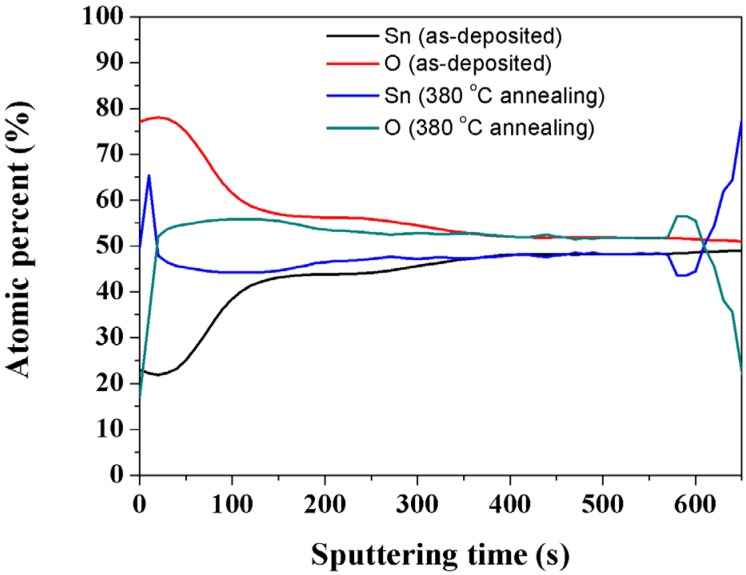
Atomic depth profile of the as-deposited and 380 °C annealing SnO_2−x_ thin films at the flow ratio of 0.5 of O_2_/(O_2_ + Ar) and a working pressure of 4.7 × 10^−2^ Pa.

Figure 6 shows the AES depth profile of as-deposited and 380 °C annealing SnO_2−x_ thin films at the flow ratio of 0.5 of O_2_/(O_2_ + Ar) and a working pressure of 4.7 × 10^−2^ Pa. The depth profile shows the change of atomic percent at the sputtering time from 0 to 120 s. The change of composition after the annealing temperature of 380 °C indicates the decrease of oxygen atoms and the increase of tin atoms in the thin film surface. The AES profiles are usually used for chemical composition analysis. We used this technique to confirm the correction for the thickness and atomic percentage with TEM.

## 3. Experimental Section

Thin films of SnO_2_ were sputtered from a diameter of 4-inch using a high purity of 99.99 wt% SnO_2_ ceramic target (Ultimate Materials Technology Co., Hsinchu, Taiwan) in an atmosphere of argon and oxygen gases having 99.999% purity. SnO_2_ layers of about 60 nm in thickness were deposited onto the glass substrates (Corning EAGLE 2000 AMLCD glass, Taichung, Taiwan) using an ion beam sputtering deposition system, the Commonwealth Scientific IBS250 Kaufman ion source, at the conditions of 600 V and 20 A, where the glass substrates were heated at 373 K. The vacuum of the ion beam sputtering chamber was maintained at 3 × 10^−4^ Pa before deposition. In the case of ion beam sputtering of SnO_2_ films, the flow ratio of mixture gas composition O_2_/(O_2_ + Ar) was controlled from 0.33 to 0.71 at a working pressure of 4.7 × 10^−2^ and 4.0 × 10^−2^ Pa during deposition. The as-deposited SnO_2_ films were subsequently annealed in Ar atmosphere from 350 °C to 400 °C for 3 h. The phase and lattice structure of the films were analyzed using grazing angle X-ray diffraction (XRD) (D/Max2500 Rigaku, Tokyo, Japan) with Cu Kα radiation (λ = 1.5406 Å) at 40 kV and 100 mA. The field-emission scanning electron microscope (FE-SEM) (S4800-I Hitachi, Tokyo, Japan) was used to observe the surface topography of the films before and after annealing at 15 kV. The cross-section morphologies of as-deposited and post-annealing films were investigated by an ultrahigh resolution transmission electron microscopy (HR-TEM) (JEM-2100F, JEOL, Peabody, MA, USA) at 200 kV, which was equipped with an energy dispersive spectrometer (EDS) INCA x-stream-2 Oxford, U.K., for chemical elemental analysis. For EDS measurements, a silicon drift detector of 80 mm^2^ was used. TEM samples were prepared using focus ion beams SMI3050SE (SEIKO, Chiba, Japan) with the first milling at 30 kV and 100 pA and the final milling at 5 kV and 40 pA. After that, Pt was coated on the SnO_2_ surface to protect thin films from damage. The Hall effect measurement setup AHM-800A with Advance Design Technology in van der Pauw configuration was used to study the electrical properties. Atomic force microscopy (AFM) (Force Precision Instrument Co., Taipei, Taiwan) was used to evaluate the surface roughness of the films by tapping mode. The sample surface was probed with a silicon tip of 10 nm in radius oscillating at its resonant frequency between 200 and 400 kHz. The scan area was 2.5 μm × 2.5 μm, and the scan rate was 0.3 Hz. The compositions and depth profile of the films were determined by the Auger Electron Spectroscopy (AES) (Microlab 350, Thermo Fisher Scientific, Warrangton, UK).

## 4. Conclusions

As-deposited SnO_2_ thin films show an amorphous structure, and crystallization occurs at 360 °C. The resistivity of the film depends strongly on the oxygen flow ratio of 0.5 and above, due to the decrease in carrier concentration. In the case of an oxygen flow ratio of 0.3, metallic ions are dominated, and stable conductivity after annealing is observed due to the change in the numbers of oxygen vacancies and excess metal ions. Therefore, the dependence of resistivity on the oxygen partial pressure could be interesting in view of the use of these materials as oxygen sensors.
